# Bacteriophage cocktail LEC2-LEC10 for broad-spectrum control of pathogenic and uncharacterized *Escherichia coli* in fresh produce

**DOI:** 10.3389/fmicb.2025.1594533

**Published:** 2025-05-19

**Authors:** Eo-Jin Kim, Sangryeol Ryu, Jeong-A Lim

**Affiliations:** ^1^Research Group of Food Safety and Distribution, Korea Food Research Institute, Wanju, Republic of Korea; ^2^Department of Food Science and Biotechnology, Chung-Ang University, Anseong, Republic of Korea; ^3^Department of Food and Animal Biotechnology, Seoul National University, Seoul, Republic of Korea; ^4^Department of Agricultural Biotechnology, Seoul National University, Seoul, Republic of Korea

**Keywords:** lytic bacteriophages, *Escherichia coli* biocontrol, phage cocktail, fresh food safety, phage receptor analysis, genome characterization

## Abstract

**Background:**

*Escherichia coli* is a major foodborne pathogen that causes intestinal diseases leading to severe illness. In particular, *E. coli* contamination in fresh produce presents a significant risk, because there are no additional sterilization processes before consumption. In this study, we characterized two novel bacteriophages, vB_EcoS_LEC2 and vB_EcoS_LEC10, and explored their use as a phage cocktail to control naturally occurring *E. coli* contamination in fresh foods.

**Methods:**

Two phages were isolated, and their antimicrobial activity and target bacterial spectrum were analyzed. The efficacy of a two-phage cocktail was evaluated against *E. coli* O157:H7 strain mixtures and naturally occurring, unidentified *E. coli* present on commercially available vegetables. The bacterial receptors recognized by the phages were identified using receptor-deficient mutants. The genome sequences of the two phages were compared, focusing on receptor-binding protein genes.

**Results:**

Characterizations revealed that both phages belonged to the *Straboviridae* family and were stable under various temperatures and pH conditions. The phages were confirmed to be strictly lytic, exhibiting short latent periods of 15 and 10 min and burst sizes of 22 and 189 phage particles per infected cell for LEC2 and LEC10, respectively. LEC2 and LEC10 exhibited distinct antimicrobial spectra, each with a broad but complementary host range among *E. coli* strains. Combining the two phages into a cocktail leveraged their complementary host specificities, broadening the overall host range and enhancing bacterial lysis against pathogenic *E. coli* mixtures compared to individual phages. The cocktail remarkably reduced the viability of naturally contaminated, unidentified *E. coli* in fresh vegetables, demonstrating its effectiveness in targeting diverse bacterial populations. LEC2 and LEC10 recognize different receptors, specifically lipopolysaccharide (LPS) (via WaaC) and OmpC, respectively, supporting their compatibility in a cocktail optimization. Furthermore, genome analysis confirmed the absence of lysogeny-related genes, toxins, and antibiotic resistance genes, reinforcing their suitability as safe biocontrol agents for food applications.

**Conclusion:**

These results demonstrate that LEC2 and LEC10, especially when used as a cocktail, are promising antibacterial agents for controlling *E. coli* contamination in fresh foods. Their complementary host ranges and strong lytic activity support their application in food safety strategies aimed at reducing *E. coli* contamination.

## 1 Introduction

*Escherichia coli*, a gram-negative bacterium belonging to the *Enterobacteriaceae* family, is commonly present in the gastrointestinal tract of mammals, including humans and animals (Tukur et al., 2014). It is a major foodborne pathogen that causes serious health conditions, including hemolytic uremic syndrome, hemorrhagic colitis, diarrhea, urinary tract infections, septicemia, and neonatal meningitis (Fan et al., 2021). Such clinical illnesses can be caused by various strains of *E. coli*, including enteroaggregative (EAEC), enteropathogenic (EPEC), enterotoxigenic (ETEC), enteroinvasive (EIEC), and enterohemorrhagic *E. coli* (EHEC) (Saxena et al., 2015). Specifically, the enterohemorrhagic *E. coli* (EHEC) serotype O157:H7 can produce harmful Shiga-like toxins, ranging from mild clinical illness to life-threatening symptoms (Puligundla and Lim, 2022).

*Escherichia coli* infections originate from various sources, such as ingestion of contaminated food products, human transmission, or direct contact with infected animals and their surroundings (Mulchandani et al., 2021). The major cause of *E. coli* outbreaks is contaminated food such as fresh produce, vegetables, and fruits (Joshi et al., 2019). With the increasing trend toward healthier lifestyles, there has been an increase in the consumption of fresh produce in its raw state (Carstens et al., 2019). However, because fresh produce is frequently consumed without a critical sterilization step in preparation, concerns arise regarding contamination throughout the production stages, such as irrigation, farming, and post-harvest processing (Feng, 2014). This becomes particularly pertinent when considering the potential for *E. coli* infections given their association with contaminated fresh produce. Currently, washing with chlorine is employed to reduce contamination but does not completely eliminate it (Kondo et al., 2006; Coulombe et al., 2020). Moreover, *E. coli* can become internalized within the lettuce stomata, forms bacterial biofilms, or survives as clusters on lettuce leaves, washing with water is not an effective way to control contamination (Luna-Guevara et al., 2019).

Bacteriophages (phages) can be used as an alternative control strategy in the food industry to reduce and inhibit pathogenic bacteria. Phages are viruses that specifically target bacteria and lyse bacterial cells by releasing progeny phage particles from the infected hosts (Ghasemian et al., 2017). Phages possess several advantageous characteristics that make them valuable as biocontrol agents. They rapidly eliminate target bacteria, self-replicate, do not infect or damage human cells, and are abundantly found in nature (Ferriol-González and Domingo-Calap, 2020). Due to their high specificity, phages selectively target bacteria, minimizing collateral damage to beneficial members of the microbiota (Schwarzer et al., 2012). These unique properties have led to the commercialization of various phage-based products, particularly in the field of food safety. Several phage products are now commercially available, having been approved as food preservatives and classified as Generally Recognized as Safe (GRAS) by the United States Food and Drug Administration (Moye et al., 2018).

Phages interact with specific ligands on their bacterial hosts through receptor-binding proteins located on their tails. The close proximity of these proteins to the tail facilitates the spatial and temporal coordination of host recognition, irreversible attachment, and genome release (Dunne et al., 2021). In some cases, the strict host specificity of phages can limit their ability to completely eradicate bacterial populations. Since individual phages have a restricted host range, a single phage may not be effective against diverse or evolving bacterial populations. To address this limitation, phage cocktails composed of multiple phages with varying host ranges have been developed to enhance pathogen control and mitigate resistance issues (Tomat et al., 2018).

In this study, we isolated two virulent phages from environmental samples that specifically target *E. coli* strains. The study aimed to characterize the biological and genetic properties of these lytic phages and assess their biocontrol potential as a cocktail against *E. coli*-contaminated fresh products under natural contamination conditions. Consequently, we investigated the effectiveness, stability, and safety of phages vB_EcoS_LEC2 and vB_EcoS_LEC10 to determine their suitability as biocontrol agents in the food industry.

## 2 Materials and methods

### 2.1 Phage isolation and phage stock preparation

Phages were isolated as described previously, with some modifications (Kim et al., 2022). Briefly, sewage samples were collected from a sewage treatment plant in Iksan and Gimje, Jeollabuk-do, South Korea, filtered, and mixed with an equal volume of 2 × Tryptic Soy Broth (TSB). *E. coli* NCCP 15961 and *E. coli* ER2738 were used as host strains for the enrichment of phages LEC2 and LEC10, respectively. Each host bacterium was inoculated into the mixture along with 2 mM MgCl_2_ and CaCl_2_, followed by incubation with shaking (180 rpm) at 37°C for 24 h. After incubation, the mixture was filtered using a 0.45-μm filter (Hyundai Micro, Seoul, South Korea) to remove residual bacterial cells, and serially diluted with sodium chloride and magnesium sulfate buffer (100 mM NaCl, 8 mM MgSO_4_∙7H_2_O, and 50 mM Tris–HCl, pH 7.5). The dilutions were spotted on each host bacteria *E. coli* NCCP 15961 and *E. coli* ER2738 overlaid plates and incubated at 37°C for 8 h to detect the presence or absence of phages in the final filtered liquid. The formed phage plaques were purified by successive pick-ups at least four times, followed by propagation through co-culture with the host strains *E. coli* NCCP 15961 and ER2738. After dialysis against sodium chloride and magnesium sulfate buffer, the phages were stored in glass vials at 4°C until further use.

### 2.2 Phage morphology

The morphology of purified phages was analyzed using transmission electron microscopy (TEM). A 20-μL aliquot of the phage stock was placed on carbon-coated copper grids and left undisturbed for 10 min. The excess sample was removed and a drop of 2% uranyl acetate (pH 4.0) was added. Immediately after staining, the excess stain was removed, the grid was air-dried for 5–10 min before TEM imaging. The morphology of the phages was examined using transmission electron microscopy (TEM; Hitachi H-7650, Japan) at Jeonbuk National University, South Korea. Phage size was measured using ImageJ software v.1.52 (National Institutes of Health, Bethesda, MD, United States).

### 2.3 Antimicrobial activity

For the bacterial challenge assay, LEC2 (10^9^ PFU/mL) and LEC10 (10^9^ PFU/mL) lysates were added to exponentially growing *E. coli* NCCP 15961 and ER2738 at multiplicity of infection (MOI) of 0.01, 0.1, 1, and 10. Cultures were incubated with shaking at 37°C, and bacterial growth was monitored over a 10-h period by measuring the absorbance at 600 nm (A600) every hour using a microplate reader (Spark^®,^ Tecan, Switzerland).

### 2.4 One-step growth curve

The phage lifecycle was analyzed using a one-step growth curve, following a previously described method (Fulgione et al., 2019) with some modifications. Phage lysates were added at a MOI of 0.001 along with 10 mM CaCl_2_ and MgCl_2_ and incubated at 37°C for 5 min to allow phage adsorption. After 5 min, the cells were collected by centrifugation and resuspended in fresh TSB. After incubation at 37°C with shaking, the bacterial culture was sampled every 5 min, and phage titer was measured using a double-layer agar method.

### 2.5 Thermal and pH stability

The thermal stability of LEC2 (10^8^ PFU/mL) and LEC10 (10^8^ PFU/mL) lysates was evaluated by incubating them at various temperatures (4, 25, 37, 50, 60, 65, and 70°C) for 60 min using a heating block. Similarly, pH stability of the phage was assessed by incubating both phage lysates for 1 h at 37°C in TSB adjusted to pH 2, 4, 6, 8, 10, and 12. All experiments were conducted using 1 mL of phage lysate in 1.75 mL microcentrifuge tubes under static conditions. After temperature and pH treatments, phage titers were determined by enumerating phage plaques.

### 2.6 Host range

The host ranges of LEC2 and LEC10 were determined using a spotting assay with the bacterial strains listed in Table 1 and Supplementary Table S1. Each strain mixed with TSB soft agar (0.4%) was overlaid on TSA (1.5%) plates, and 10 μL of phage lysates (10^9^ PFU/mL) were spotted onto the solidified surface. The presence of a clear zone at the site of phage application (indicating no turbidity) signified strong lytic activity, whereas the absence of a clear zone (complete turbidity) indicated no lytic activity.

**Table 1:** Host range of LEC2 and LEC10 against *E. coli* strains.

Bacterial strains				Phages		
				LEC2	LEC10	Cocktail
1	*E. coli*	DH5α		**+++**	**+++**	**+++**
2	*E. coli*	OE50	O157:H7	**+**	**+**	**++**
3	*E. coli*	NCTC 12079	O157:H7	**+**	**+**	**+**
4	*E. coli*	NCCP 13937	O26	**++**	**+++**	**+++**
5	*E. coli*	DH10B		**+++**	**+++**	**+++**
6	*E. coli*	BL21(DE3)		**+++**	**−**	**+++**
7	*E. coli*	ER2738		**+++**	**+++**	**+++**
8	*E. coli*	ATCC 47000		**++**	**++**	**+++**
9	*E. coli*	ATCC 10536		**+**	**+**	**+++**
10	*E. coli*	ATCC 9637		**++**	**+**	**+++**
11	*E. coli*	ATCC 11775	O1:K1:H7	**+**	**+**	**++**
12	*E. coli*	ATCC 43890	O157:H7	**+**	**++**	**+**
13	*E. coli*	ATCC 43889	O157:H7	**+**	**+**	**+**
14	*E. coli*	ATCC 43894	O157:H7	**+**	**++**	**+**
15	*E. coli*	ATCC 35150	O157:H7	**+**	**+**	**+**
16	*E. coli*	NCCP 11091	O157:H7	**+**	**+**	**+**
17	*E. coli*	ATCC 43895	O157:H7	**+**	**+**	**++**
18	*E. coli*	NCCP 14540	O111	**−**	**+**	**+**
19	*E. coli*	NCCP 12537	O103	**+**	**−**	**++**
20	*E. coli*	NCCP 12551	O121	**+**	**+**	**+**
21	*E. coli*	NCCP 13581	O111	**−**	**+**	**++**
22	*E. coli*	NCCP 13667	O26	**+++**	**+++**	**+++**
23	*E. coli*	NCCP 13721	O104	**−**	**++**	**+++**
24	*E. coli*	NCCP 14010	O159	**+**	**−**	**+**
25	*E. coli*	NCCP 14020	O26	**+++**	**+**	**++**
26	*E. coli*	NCCP 14538	O157	**+**	**++**	**++**
27	*E. coli*	NCCP 15647	O11	**+**	**−**	**+**
28	*E. coli*	NCCP 15656	O104	**−**	**++**	**+++**
29	*E. coli*	NCCP 15739	O157:H7	**+**	**+**	**++**
30	*E. coli*	NCCP 15954	O145	**+**	**+**	**+++**
31	*E. coli*	NCCP 15956	O103	**+**	**−**	**++**
32	*E. coli*	NCCP 15961	O26	**++**	**+**	**+++**
33	*E. coli*	ATCC 25922		**+**	**−**	**++**
34	*E. coli*	ATCC 8739		**+**	**+**	**+++**

+++, clear plaque; ++, semi-clear plaque; +, turbid plaque.

### 2.7 Lytic activity of the phage cocktail against pathogenic *Escherichia coli* mixture

Phage cocktails were prepared by mixing equal volumes of LEC2 (10^9^ PFU/mL) and LEC10 (10^9^ PFU/mL). Five pathogenic *E. coli* strains (ATCC 43890, ATCC 43894, NCCP 14538, NCCP 15956, and NCCP 15961) were selected based on their clinical relevance and host range diversity. Each strain was grown individually to the exponential phase, and then combined in equal volumes to create a mixed culture. To evaluate the effects of individual phages and the phage cocktail in liquid culture, individual phage lysates and the cocktail were added to an exponentially growing *E. coli* mixture at an approximate multiplicity of infection (MOI) of 0.1. Bacterial growth was monitored over a 10-h incubation period by measuring absorbance at 600 nm (A_600_) every hour using a microplate reader (Spark^®^, Tecan, Switzerland).

### 2.8 Phage cocktail to control naturally occurring *Escherichia coli* in fresh vegetables

Twenty fresh vegetables, including leafy salads, lettuce, and sprouted vegetables, were purchased from a local supermarket in Jeonju, South Korea. For each vegetable, a 30 g sample was taken and homogenized with 50 mL of TSB in a sample cup to ensure uniform distribution. From the resulting mixture, two 5 mL aliquots were separately transferred into individual tubes to create paired experimental samples. To one tube, 0.6 mL of a phage cocktail (prepared by combining LEC2 and LEC10 phage lysates at a 1:1 ratio, 10^9^ PFU/mL) was added, while the other tube received 0.6 mL of TSB as a negative control. This setup was designed to ensure that both treated and control samples originated from an identical initial mixture, minimizing variability prior to phage treatment. This process was repeated twice per vegetable. After phage or control treatment, the samples were incubated at 37°C for 2 h, a condition chosen to maximize phage lytic activity under unknown contamination status. Following incubation, the medium was serially diluted 10-fold, and 1 mL of each dilution was spotted onto 3 M Petrifilm™ *E. coli*/coliform Count Plates (EC). After 24 h incubation at 37°C, only colonies identified as *E. coli* based on morphology (i.e., black dots with a surrounding blue halo) were counted. This selective and differential medium allows the growth of only *E. coli* and coliform bacteria, enabling reliable enumeration of *E. coli* in the samples.

### 2.9 Bacterial receptors for phage binding

The *E. coli* MG1655 strain was used to analyze host receptors for phage LEC2 and LEC10. Based on previous research, key genes related to phage receptors in *E. coli* MG1655 were selected and analyzed (Hantke, 2020; Shin et al., 2024). To identify the receptors of the two phages, a spotting assay was performed using the wild-type (WT) *E. coli* MG1655 strain and mutant strains lacking specific receptor related genes, including *E. coli* MG1655 Δ*btuB*, Δ*fadL*, Δ*fhuA*, Δ*fliC*, Δ*lamB*, Δ*ompA*, Δ*ompC*, Δ*ompF*, Δ*tolC*, Δ*waaC*, and Δ*waaG*. The mutant strains used in this study were provided by the Molecular Food Microbiology Laboratory at Seoul National University.

Complementation of the target genes (*waaC* and *ompC*) in the deletion mutants *E. coli* MG1655 Δ*waaC* and Δ*ompC* was carried out by cloning the corresponding genes into the plasmid pUC19. The *waaC* and *ompC* genes of *E. coli* MG1655 were amplified using the primers waaC_F_HindIII, waaC_R_EcoRI, ompC_F_HindIII, and ompC_R_KpnI (Table 2). The PCR product for *waaC* was digested with *Hind*III and *Eco*RI, and ligated into *Hind*III/*Eco*RI-predigested pUC19. Similarly, for *ompC*, *Hind*III, and *Kpn*I restriction enzymes were used for digestion and ligation. The ligated DNA was transformed into *E. coli* DH5α to construct the recombinant plasmid. The recombinant plasmids were then introduced into the deletion mutants by electroporation, and the transformants were selected on ampicillin plates (100 μg/mL). Finally, *waaC* and *ompC* gene expression was induced with 1 mM Isopropyl β-D-1-thiogalactopyranoside (IPTG).

**Table 2:** Primers used for cloning in receptor complementation assays.

Primer name	Sequences (5′ to 3′)
waaC_F_HindIII	TAA AAA AGC TTA TAA AGG CAT ATA ACA
waaC_R_EcoRI	TTT GTT CGG TAC CAA TCG AGA
ompC_F_HindIII	ATC ACC AAA GCT TAA TCG AC
ompC_R_KpnI	CAT TGA TGA ATT CAG AGT GTA A

### 2.10 Genome sequencing and bioinformatics analysis

Phage DNA was extracted from a high-titer phage stock (10^10^ PFU/mL) using the Phage DNA Isolation Kit (Norgen Biotek, ON, Canada) (Naveen Kumar et al., 2024). The extracted phage DNA was sequenced using the Illumina MiSeq platform (Illumina, San Diego, CA, United States), and *de novo* assembly of the reads was performed using SPAdes v.3.13.0 (Prjibelski et al., 2020) at Sanigen Co. Ltd., South Korea. The phage nucleotide sequences were analyzed using BLASTN at the National Center for Biotechnology Information (NCBI). The open reading frames (ORFs) were predicted using RAST Server (Aziz et al., 2008; Salamov and Solovyevand, 2011) and annotated based on the functions of putative gene products using the NCBI online tool BLASTP^1^ (Altschul et al., 1997). Antibiotic resistance and virulence factor genes were screened using the CARD (comprehensive antibiotic resistance database)^2^ and UniProt,^3^ respectively. The CGview Server was used to generate a phage genome map (Li et al., 2021). Genomic comparisons were performed using Easyfig v2.2.2^4^ (Sullivan et al., 2011).

## 3 Results and discussion

### 3.1 Isolation and morphological characterization

**Figure 1:**
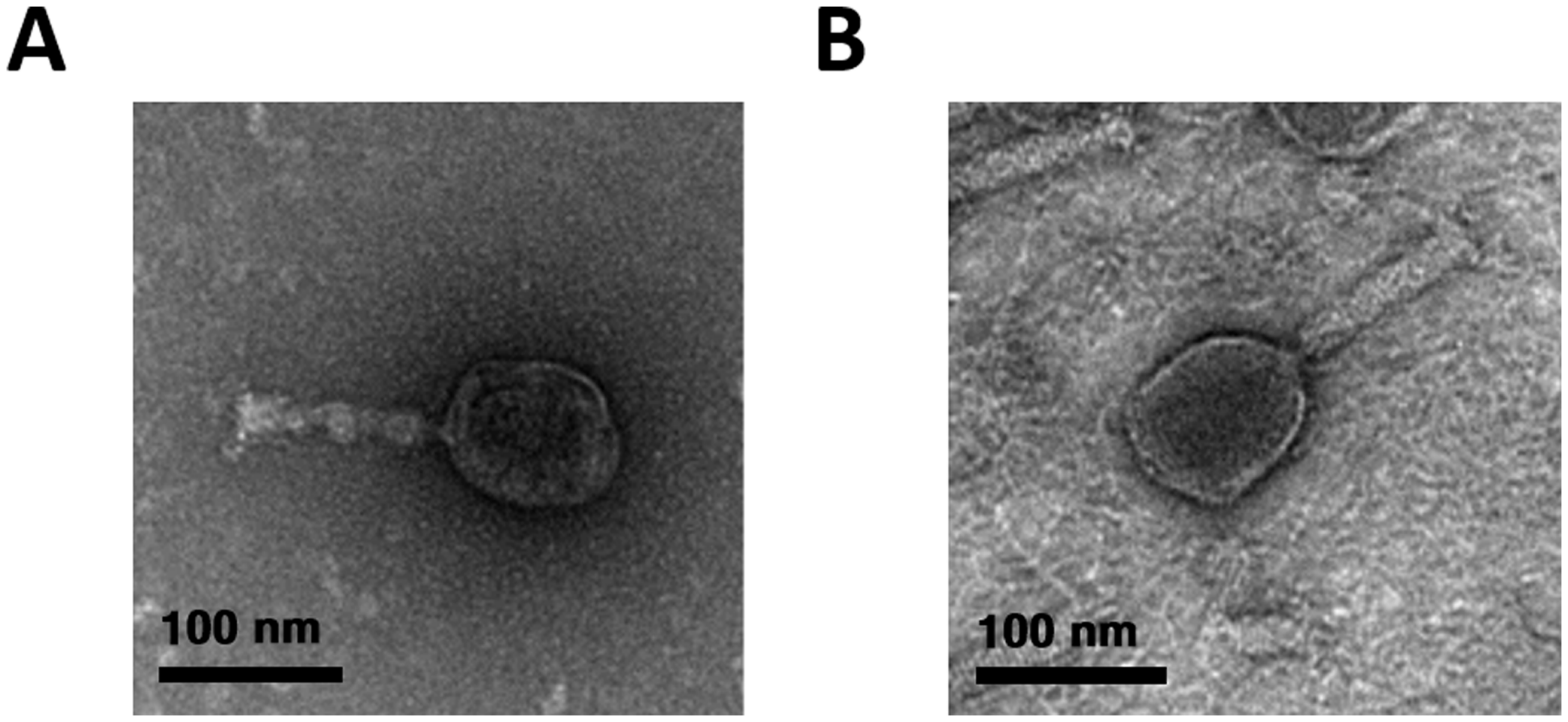
Morphological characterization of phages LEC2 **(A)** and LEC10 **(B)** by transmission electron microscopy (TEM). Both phages exhibited icosahedral heads with long contractile tails, consistent with typical myovirus morphology. Scale bars represent 100 nm.

Phages were isolated from a sewage sample using *E. coli* NCCP 15961 and ER2738 as host strains. The isolated phages, vB_EcoS_LEC2 and vB_EcoS_LEC10, produced clear plaques on the lawns of their respective host bacteria. Morphological characterization using transmission electron microscopy (TEM) revealed that both LEC2 and LEC10 had icosahedral heads and long tails (Figure 1). Phage LEC2 possessed an icosahedral head measuring 96.6 ± 2.4 nm in length, accompanied by a tail of 102.1 ± 2.0 nm in length (*n* = 5). Similarly, phage LEC10 exhibited an icosahedral head with a length of 97.2 ± 1.4 nm and a tail measuring 106.4 ± 1.1 nm (*n* = 5). Further genomic analysis classified the phages within the *Caudoviricetes* order *Straboviridae* family, with LEC2 assigned to the genus *Tequatrovirus* and LEC10 to the genus *Mosigvirus*, demonstrating concordance between morphological and genomic classifications (Zhu et al., 2024).

### 3.2 Antimicrobial activity and one-step growth characteristics

**Figure 2:**
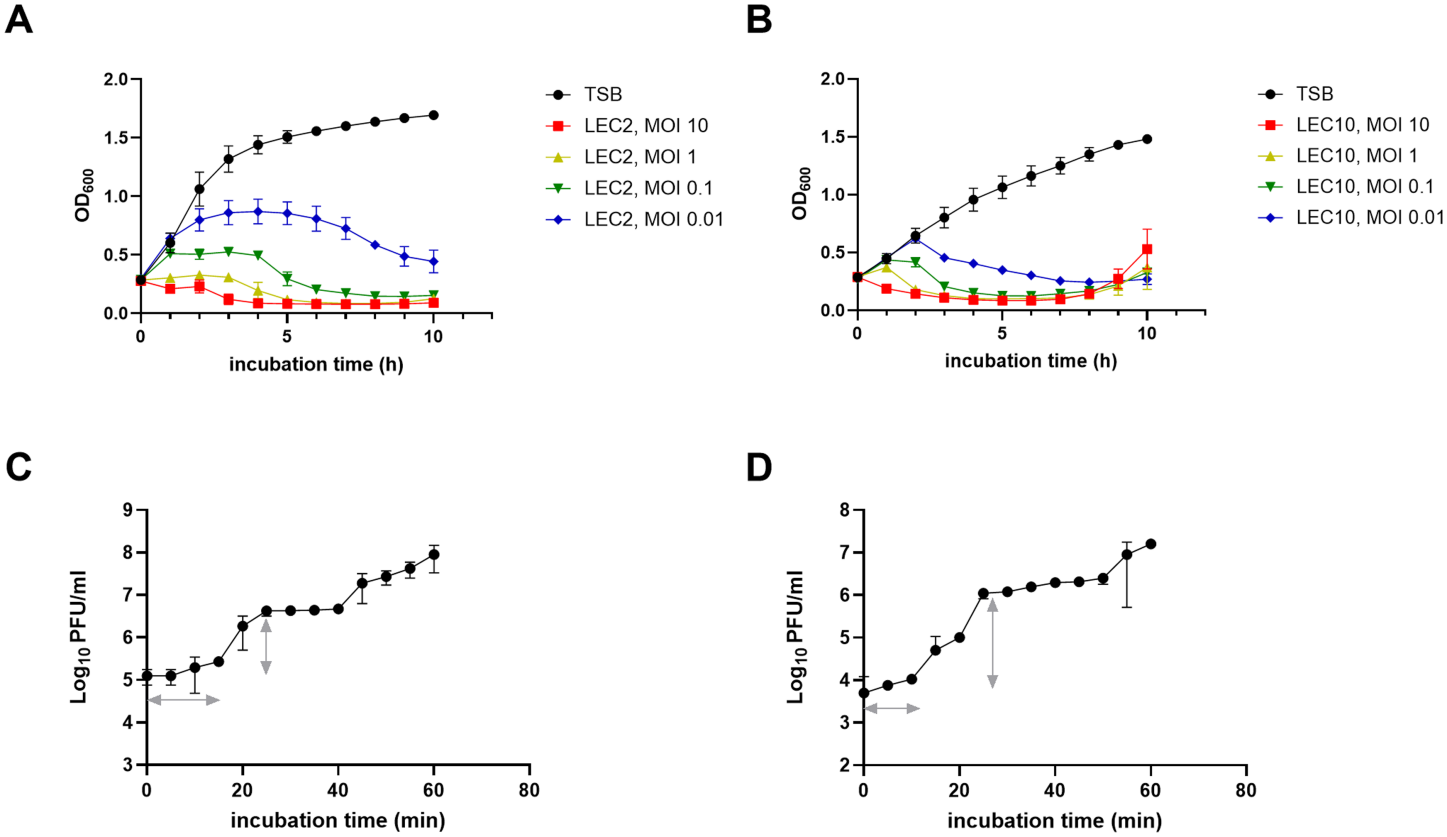
Antimicrobial activity and one-step growth characteristics of phages LEC2 and LEC10. **(A,B)** Bacterial growth curves showing antimicrobial activity of LEC2 against *E. coli* NCCP 15961 **(A)** and LEC10 against *E. coli* ER2738 **(B)** at various MOIs. **(C,D)** One-step growth curves illustrating the latent period and burst size of LEC2 **(C)** and LEC10 **(D)**. Data points represent mean ± SD from three independent experiments.

The lytic activity of the isolated phages was assessed by evaluating bacterial growth suppression at different MOIs (0.01, 0.1, 1, and 10). LEC2 and LEC10 effectively lysed *E. coli* NCCP 15961 and ER2738 at all tested MOIs for up to 10 h (Figures 2A,B). Initially, bacterial growth increased steadily in the control groups and at lower MOIs; however, as phage activity intensified in an MOI-dependent manner, bacterial growth was gradually suppressed. At MOI of 10 and 1, both phages strongly inhibited bacterial growth from the beginning of incubation, maintaining OD_600_ values close to zero for up to 10 h. At lower MOIs (0.1 and 0.01), bacterial control was less effective, likely due to an insufficient phage-to-host ratio, allowing bacterial escape or resistance mechanisms. However, even at these lower MOIs, OD_600_ values gradually decreased with prolonged incubation, indicating that antimicrobial effects were still observed, albeit with a delayed onset of bacterial lysis (Figures 2A,B).

One-step growth curve were analyzed to determine the latent period (time before the first phage progeny are released) and burst size (number of phage particles produced per infected cell). LEC2 exhibited a latent period of 15 min, with a burst size of 22 PFU per infected *E. coli* cell (Figure 2C). In contrast, LEC10 demonstrated rapid intracellular replication and assembly, completing a shorter latent period of 10 min and a significantly larger burst size of approximately 189 PFU per infected cell (Figure 2D). Notably, the burst size of LEC10 was considerably larger than that of other *Straboviridae E. coli* phages (50–100 PFU/cell) reported in previous studies (Litt and Jaroni, 2017; Lu et al., 2022). Phages with short latent periods and high burst sizes are preferred for food biocontrol applications (Li et al., 2023). While LEC2 may not exhibit as much biocontrol potential as LEC10 based on burst size and latent period alone, its host specificity and potential synergistic effects within a phage cocktail warrant further investigation.

### 3.3 Thermal and pH stability

**Figure 3:**
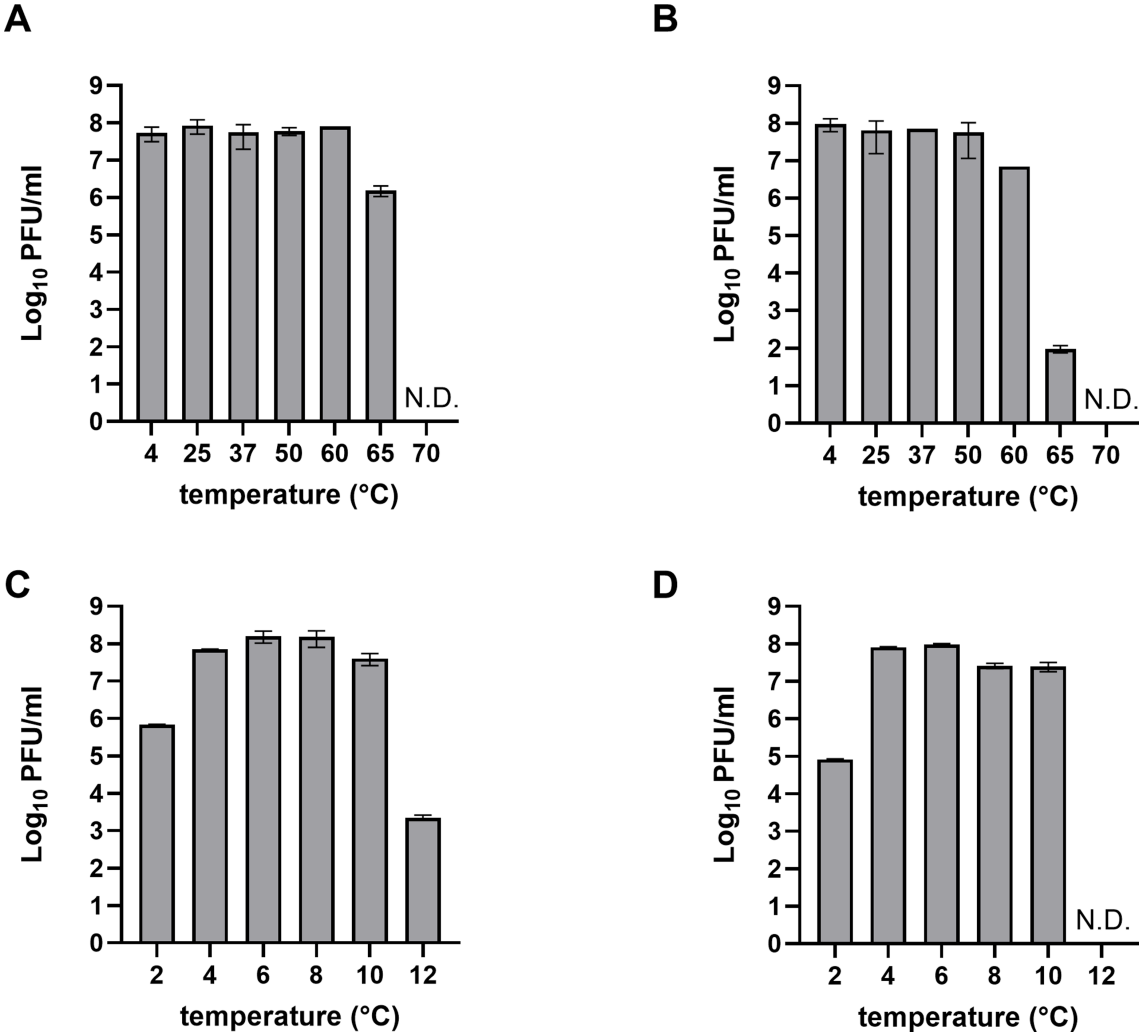
Thermal and pH stability of phages LEC2 and LEC10. Thermal stability of LEC2 **(A)** and LEC10 **(B)** at various temperatures (−4 to 70°C). Stability of LEC2 **(C)** and LEC10 **(D)** at pH values ranging from 2 to 12. Data points represent mean ± SD from three independent experiments.

For the application of phages in food, their stability must be confirmed under various environmental conditions, including different temperatures and pH levels (Lewis and Hill, 2020). The stability of LEC2 and LEC10 was evaluated across a broad range of temperatures (−4 to 70°C) and pH values (2 to 12). Both LEC2 and LEC10 remained stable between 4°C and 50°C, with LEC2 retaining stability even at 60°C. However, at 65°C and above, phage titers gradually declined, and no plaques were detected at 70°C (Figures 3A,B). Similarly, both phages were stable under a wide range of pH (4–10) (Figures 3C,D). Under strongly acidic conditions (pH 2), phage activity decreased; however, many phages retained their ability to infect bacteria. Notably, even at pH 12, where most previously studied phages were completely inactivated, LEC2 maintained partial activity, demonstrating exceptional stability (Kitti et al., 2022; Zhu et al., 2022). These findings suggest that LEC2 and LEC10 have strong potential for the biocontrol of foodborne pathogens, as they remain stable under a range of environmental conditions, including varying pH levels, temperatures, and food processing conditions.

### 3.4 Host range

Generally, phages exhibit high specificity toward their target bacteria. Since phages LEC2 and LEC10 were isolated using *E. coli* as their host strains, it was expected that they would exhibit *E. coli*-specific activity. To experimentally confirm their host specificity, we evaluated their lytic activity against various major foodborne pathogenic bacteria, including *Bacillus cereus*, *Cronobacter sakazakii*, *Listeria monocytogenes*, *Salmonella* spp., *Staphylococcus aureus*, and *Yersinia enterocolitica* (Supplementary Table S1). As anticipated, neither LEC2 nor LEC10 displayed lytic activity against any of these tested non-*E. coli* strains, verifying their specificity toward *E. coli*. Broad lytic activity of phages against non-target bacteria can lead to unintended consequences, such as the emergence of phage-resistant strains or disruption of existing microbial communities. In this regard, the absence of such activity by our phage cocktail highlights its desirable specificity and supports its potential as a safe biocontrol agent. Next, we examined the host range of phages LEC2 and LEC10 more comprehensively using 34 different *E. coli* strains, including 17 pathogenic and 17 non-pathogenic strains (Table 1). The lytic activity was assessed by spotting phage lysates onto the lawns of the target *E. coli* strains and observing the formation of lysis zones and their clarity. The results showed that phages LEC2 and LEC10 exhibited a broad host range within *E. coli*, lysing 30 out of 34 (88%) and 28 out of 34 (82%) *E. coli* strains across different serotypes, respectively (Table 1). These included several NCCP strains that were originally isolated from clinical cases, indicating that the phage cocktail is effective not only against laboratory reference strains but also against strains derived from real infection sources. This observation of broad host range is consistent with previous research on the tendency of *Straboviridae* phages to have a broader host range compared to other phage families (Sørensen et al., 2021; Liao et al., 2024).

Importantly, LEC2 and LEC10 exhibited complementary lytic activity against the tested *E. coli* strains. Each phage was able to lyse strains that the other could not, ensuring broader host coverage. For instance, four strains (*E. coli* NCCP 14540, NCCP 13581, NCCP 13721, and NCCP 15656) that were resistant to LEC2 alone were successfully lysed by the LEC2-LEC10 phage cocktail, leading to plaque formation. Similarly, LEC10 alone was ineffective against certain strains that were susceptible to LEC2. As a result, the cocktail approach enabled complete lysis of all tested *E. coli* strains (Table 1). These findings highlight the effectiveness of combining phages with complementary host ranges to achieve broader bacterial control. The LEC2-LEC10 cocktail demonstrates strong potential as a biocontrol strategy, effectively targeting diverse *E. coli* strains, including naturally contaminated or unidentified *E. coli* in food (Oh et al., 2024).

### 3.5 Control of pathogenic *Escherichia coli* mixture by phage cocktail

To assess the bacterial suppression capability of the phage cocktail, individual phages and the phage cocktail were added to a mixture of five STEC strains that were arbitrarily selected from a subset of clinically relevant isolates representing diverse serogroups, and the differences in the inhibitory efficacy were compared. Among diverse *E. coli* strains, pathogenic *E. coli* is responsible for enteric diseases, including abdominal cramps, diarrhea, and vomiting (Hunt, 2010). In particular, Shiga toxin-producing *E. coli* (STEC) is associated with severe foodborne outbreaks and poses significant public health concerns (Mangieri et al., 2020). Given the importance of STEC, strains representing four major serogroups (O157:H7, O157, O103, and O26) were first selected for inclusion, and five representative isolates were then arbitrarily chosen for analysis: ATCC 43890, ATCC 43894 (both O157:H7), NCCP 14538 (O157), NCCP 15956 (O103), and NCCP 15961 (O26).

**Figure 4:**
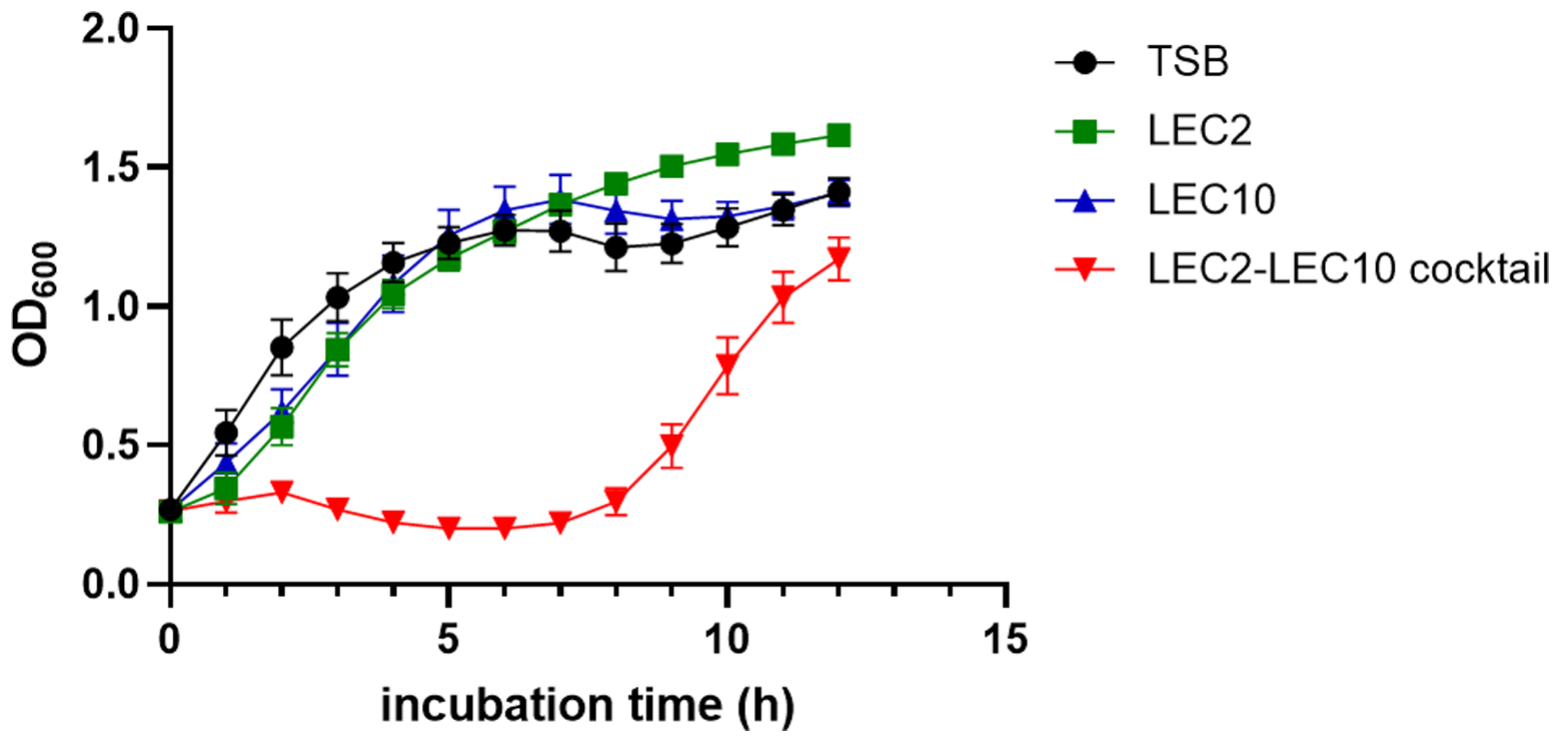
Bacterial challenge assay assessing the antimicrobial efficacy of individual phages (LEC2, LEC10) and the phage cocktail against a pathogenic *E. coli* mixture. Bacterial growth was monitored by measuring optical density (OD₆₀₀) over a 10-h incubation period. The phage cocktail exhibited significantly enhanced inhibitory effects compared to individual phage treatments. TSB served as the untreated control.

According to the host range data presented in Table 1, the five selected *E. coli* strains exhibited differential susceptibility to phages LEC2 and LEC10 (Table 1). Specifically, each individual phage failed to lyse at least one strain in the mixture, whereas all five strains were susceptible to at least one of the two phages. This experimental design enabled a direct assessment of whether combining the two phages could compensate for their respective host range limitations. As expected, the phage cocktail demonstrated superior bactericidal activity against the pathogenic *E. coli* mixture compared to individual phages. The limited inhibitory effect observed with individual phages is attributable to their inability to target all five strains, as evidenced by the progressive increase in OD₆₀₀ values over time. When each individual phage was added to the bacterial mixture, the growth of bacteria steadily increased from the early stages of cultivation reaching OD₆₀₀ values of 1.6 and 1.4, respectively, after 10 h of incubation, no difference compared to the negative control (without phage). In contrast, upon the combination of phages into a cocktail, significant growth inhibition was observed with OD₆₀₀ values remained below 0.2 for up to 7 h of incubation, indicating effective control over bacterial growth (Figure 4).

Despite the initial suppression of bacterial growth by the phage cocktail, an increase in OD_600_ was observed over time, likely due to the emergence of resistant bacterial populations (Gurney et al., 2020). The growth of resistant strains can be addressed by employing a greater variety of phage combinations (Gvaladze et al., 2024).

### 3.6 Control of naturally occurring *Escherichia coli* in fresh vegetables by phage cocktail

**Figure 5:**
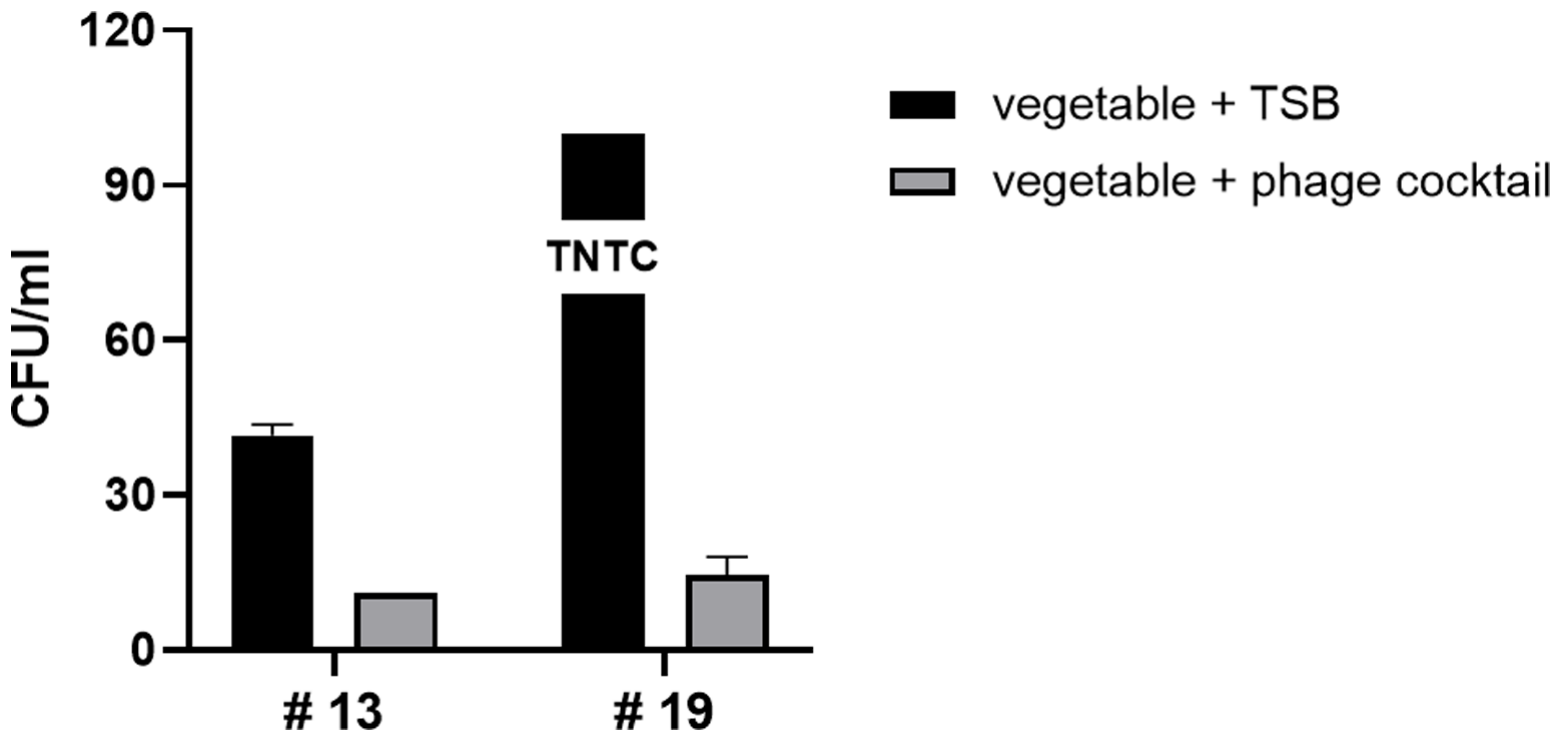
Efficacy of the phage cocktail against naturally occurring *E. coli* on fresh vegetables. Two vegetables (#13 and #19) naturally contaminated with unidentified *E. coli* strains were treated with the LEC2-LEC10 phage cocktail. The bacterial counts (CFU/mL) after phage cocktail treatment (gray bars) were substantially reduced compared to the untreated control (TSB only, black bars). Eighteen other tested vegetables showed no detectable contamination and are not shown.

Next, we evaluated the biocontrol potential of the LEC2 and LEC10 phage cocktail on fresh vegetables with unknown levels of naturally occurring *E. coli* contamination. Twenty fresh vegetables, including leafy salads, lettuce, and sprouted vegetables, were purchased from a local supermarket. Among these, *E. coli* contamination was found in two vegetables (#13, #19), while the remaining 18 showed no contamination (Figure 5). Treatment with the phage cocktail greatly reduced these unidentified environmental *E. coli* strains. In #13, although *E. coli* was detected at a relatively low count of 42 CFU/mL, phage cocktail application led to a substantial reduction of the bacterial population, leaving only a minimal amount after phage treatment. Notably, in #19, where *E. coli* contamination was too numerous to count, the phage cocktail demonstrated maximal inhibitory effects, leading to the substantial reduction of the majority of the bacterial population (Figure 5). Since the detected *E. coli* strains were unidentified, their pathogenic potential remained unknown. However, the ability of the phage cocktail to eliminate a broad spectrum of naturally occurring *E. coli* suggests its potential to also target pathogenic strains that may be present within the microbial population. This highlights the applicability of the phage cocktail beyond controlled laboratory settings, as it effectively reduced *E. coli* from fresh produce under real-world conditions. These results confirm the effectiveness of phages as biocontrol agents, demonstrating that the LEC2-LEC10 phage cocktail effectively targets diverse and unidentified *E. coli* strains in fresh produce, potentially including pathogenic variants. Despite the limited number of phages in the cocktail, each phage exhibited a sufficiently broad and complementary host range, allowing for robust biocontrol efficacy.

Although the phage cocktail significantly reduced the population of unidentified *E. coli*, trace amounts persisted. To eliminate these residual *E. coli*, higher MOI phage treatment or supplementation with additional phages exhibiting diverse host ranges may be required. This approach aligns with our previous result (Figure 2), which demonstrates that increasing phage concentration improves antimicrobial efficiency. Previous studies have also shown that enhanced bactericidal effects can be achieved by either increasing phage concentration (Lewis and Hill, 2020) or incorporating a more diverse phage cocktail (Korf et al., 2020; Erol and Keskin, 2024).

### 3.7 Identification of host receptors

The host range of a phage is determined by the specific receptor that the phage recognizes on the surface of its host bacterium (Shin et al., 2012). These receptors may include outer membrane proteins, flagella proteins, or lipopolysaccharides (LPS) (Gambino and Sørensen, 2024). Optimizing a phage cocktail that targets multiple receptors increases the likelihood of effective bacterial control by broadening target recognition (Stone et al., 2019). In this study, we first observed that phages LEC2 and LEC10 exhibited distinct host ranges, suggesting differences in their receptor recognition. To confirm the molecular basis of their host range differences, we subsequently identified the specific receptors utilized by each phage.

**Figure 6:**
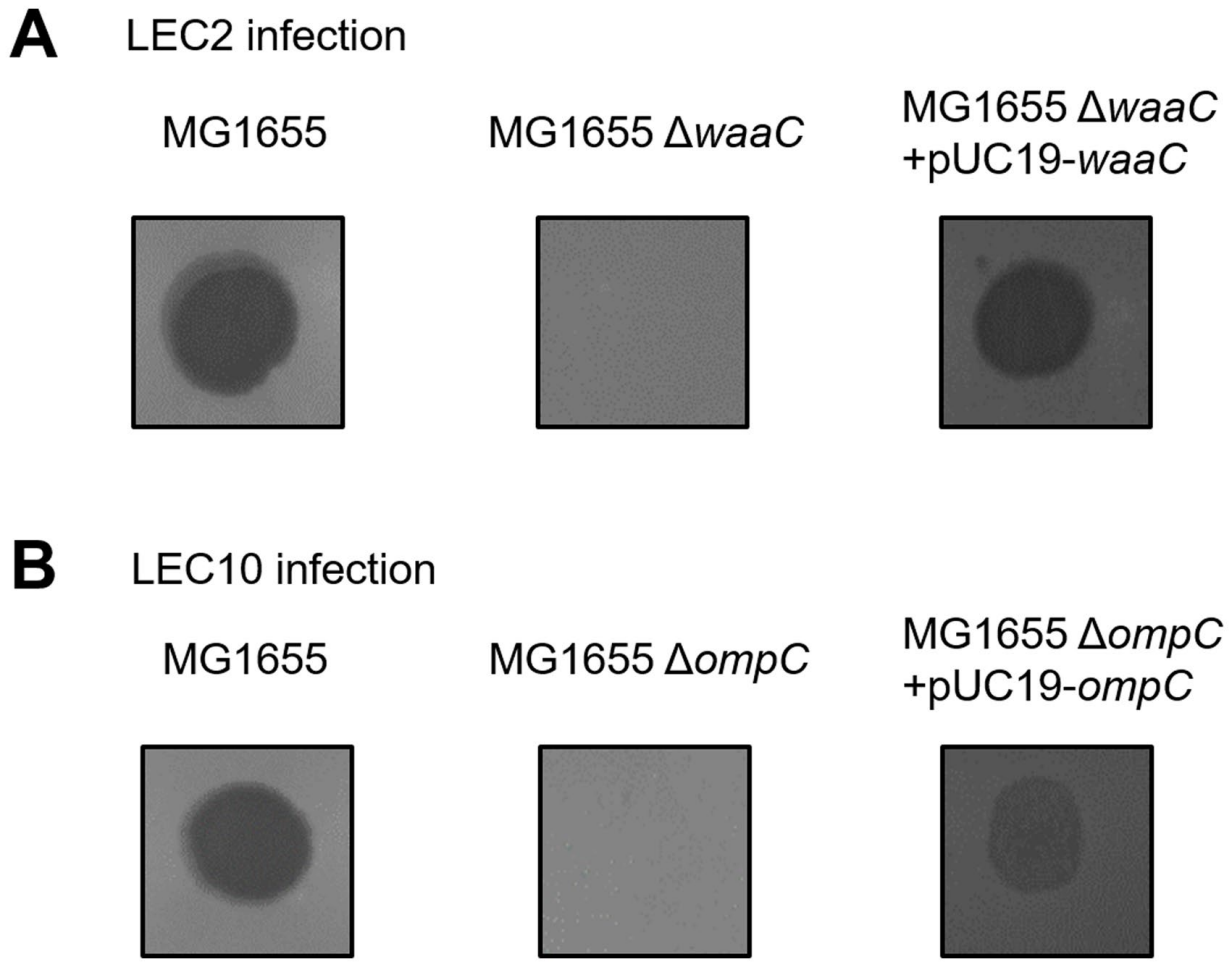
Identification of host receptors for phages LEC2 and LEC10. **(A)** Phage LEC2 failed to infect *E. coli* MG1655 Δ*waaC* mutant but successfully infected the complemented strain (Δ*waaC* with pUC19-*waaC*). **(B)** Phage LEC10 failed to infect the Δ*ompC* mutant but successfully infected the complemented strain (Δ*ompC* with pUC19-*ompC*). Wild-type *E. coli* MG1655 served as a positive control.

To identify the host receptors used by phages LEC2 and LEC10, we evaluated their ability to infect and lyse wild-type *E. coli* MG1655 and various receptor-deficient mutants. The mutant strains contained deletions in genes related to key receptors, including *btuB* (vitamin B12 uptake protein), *fadL* (long-chain fatty acid transport protein), *fhuA* (outer membrane transport protein), *fliC* (flagellin), *lamB* (maltoporin), *ompA* (outer membrane protein A), *ompC* (outer membrane protein C), *ompF* (outer membrane protein F), *tolC* (outer membrane protein TolC), *waaC* (HepI transferase, involved in LPS inner core biosynthesis), and *waaG* (GlcI transferase, involved in LPS outer core biosynthesis) (Hantke, 2020; Shin et al., 2024). Phages LEC2 and LEC10 failed to form plaques on the lawns of *E. coli* MG1655 Δ*waaC* and Δ*ompC*, respectively (Figure 6). Gene complementation assays were performed by cloning the *waaC* and *ompC* genes into the expression vector pUC19 and transforming them into their respective deletion mutants. Phage infection was restored in the complemented strains (Figure 6), as evidenced by the appearance of lysis zones, confirming that LPS and OmpC serve as the receptors for LEC2 and LEC10, respectively.

The *waaC* gene encodes HepI transferase, which catalyzes the addition of L-glycero-D-manno-heptose (heptose) to the Kdo (3-deoxy-D-manno-oct-2-ulosonic acid) region of the LPS inner core (Piya et al., 2020; Han et al., 2024). This enzymatic step is essential for proper LPS structure, impacting bacterial outer membrane stability and phage susceptibility (Washizaki et al., 2016). Many phages, particularly those with myovirus morphotypes, recognize LPS as a primary receptor (Nobrega et al., 2018). Our findings (Figure 6) align with previous studies demonstrating that LPS modifications or deficiencies can drastically alter phage sensitivity. Given the essential role of LPS in *E. coli*, it is likely that phages such as LEC2 have evolved to target this ubiquitous structure to ensure efficient host recognition and infection.

Notably, phage LEC2 was unable to infect the Δ*waaC* mutant but retained infectivity toward the Δ*waaG* mutant. Both *waaC* and *waaG* are involved in LPS core biosynthesis; however, their roles differ. *waaC* is required for heptose addition to the Kdo region in the LPS inner core, while *waaG* encodes a glucosyltransferase that adds glucose to the heptose II residue (Yethon et al., 2000). A mutation in *waaG* results in LPS structure truncation after the inner core heptose residues, yet the inner core itself remains largely intact. In our study, phage LEC2 was unable to infect the Δ*waaC* mutant but retained infectivity toward the Δ*waaG* mutant. This suggests that LEC2 requires the complete inner core structure of LPS, as synthesized by WaaC, for successful infection. The ability of LEC2 to infect the Δ*waaG* mutant indicates that the glucose I residue added by WaaG is not essential for its receptor recognition. Therefore, LEC2 likely recognizes the heptose residues within the inner core of LPS as its receptor.

Meanwhile, phage LEC10 was found to use OmpC as its receptor. OmpC, a major outer membrane porin in *E. coli*, facilitates the passive diffusion of small molecules and serves as a receptor for phages (Washizaki et al., 2016; Chen et al., 2020). The absence of OmpC in the mutant strain markedly reduced LEC10 infectivity, confirming its role as a critical receptor.

By identifying the specific receptors of each phage, we confirmed that the host range differences between LEC2 and LEC10, initially observed across multiple *E. coli* strains, were due to distinct receptor recognition. This validation further supports the rationale for optimizing a phage cocktail, demonstrating that combining LEC2 and LEC10 was an effective strategy for broadening bacterial targeting.

### 3.8 Genomic characterization and comparative genome analysis

#### 3.8.1 Genomic characterization

**Figure 7:**
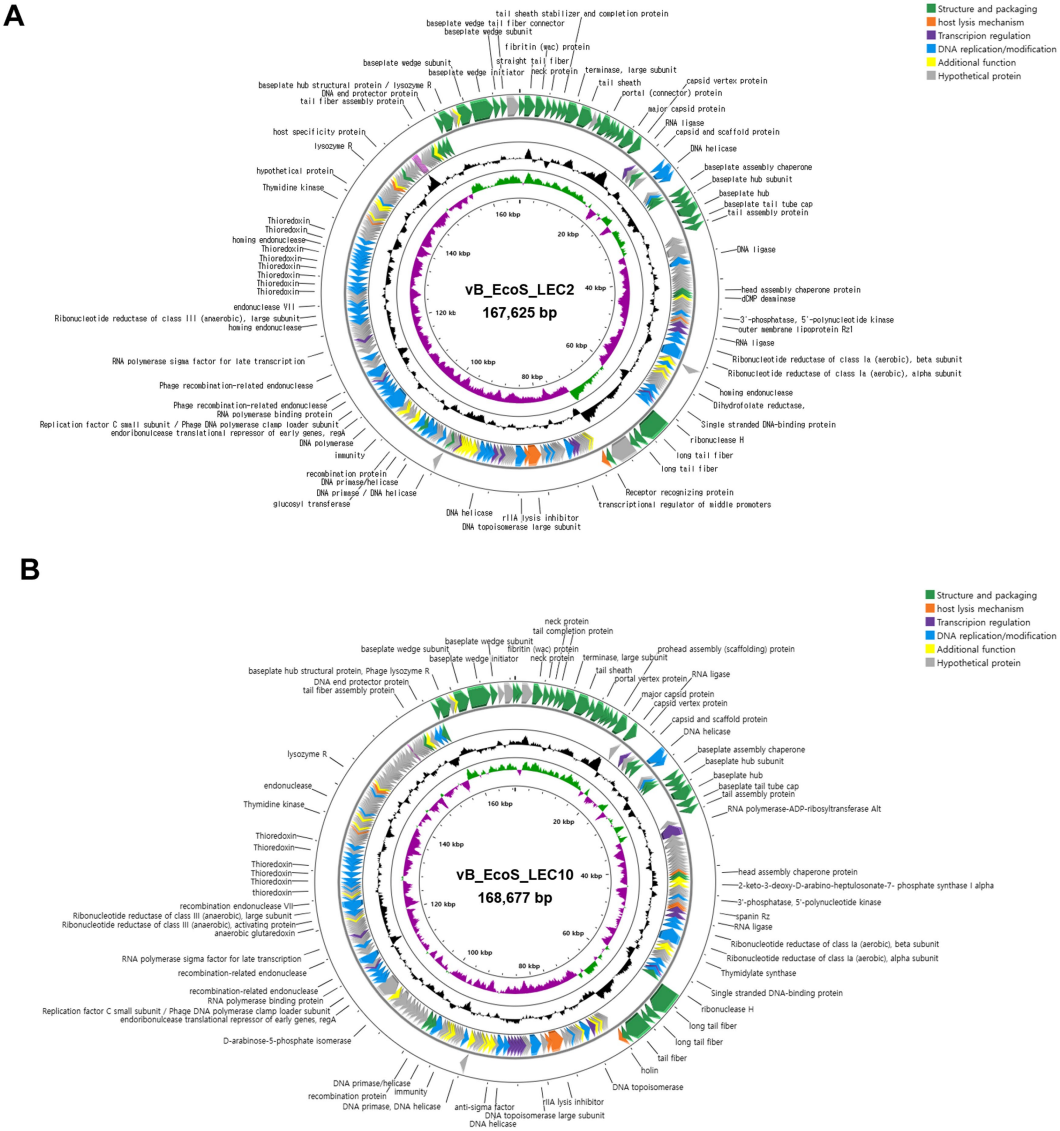
Annotated genome maps of phages. Circular genome maps of phages LEC2 **(A)** and LEC10 **(B)**, highlighting predicted open reading frames (ORFs) grouped by functional categories. Arrows indicate the direction of transcription, and functional annotations include structure and packaging, host lysis, transcriptional regulation, DNA replication/modification, and other additional functions.

To understand phage infection and host-phage interactions at the molecular level, the complete genome sequences of LEC2 and LEC10 were determined. Analysis revealed that phage LEC2 genome consisted of 167,474 bp with a GC content of 35.31%, containing 269 predicted ORFs and 11 tRNA genes (Supplementary Table S2). Similarly, the LEC10 genome consisted of 168,677 bp with a GC content of 37.56%, containing 267 predicted ORFs and two tRNA genes (Supplementary Table S3). Among the predicted ORFs, 142 (52.8%) in LEC2 and 128 (47.9%) in LEC10 were assigned specific functions (Figure 7). These ORFs were categorized into five functional groups: (1) structure and packaging (major capsid protein, baseplate wedge, neck protein, and internal head protein in short tail fibers, tail sheath proteins, long tail fibers, and receptor recognition proteins); (2) host lysis (endolysin and holin); (3) transcriptional regulation (transcription regulator MotB, RNA polymerase binding protein, sigma factor, and ADP-ribosylase); (4) DNA replication/modification (DNA helicase, primase, and polymerase); and (5) additional functions (Figure 7). Notably, lysogeny-related genes, such as integrase, transposase, repressor, and genome attachment site (*attP*), were absent in both LEC2 and LEC10 genomes, suggesting that they are strictly lytic phages (Zamora-Caballero et al., 2024). This was further supported by life cycle prediction using the PhageAI tool,^5^ which classified both phages as lytic (Tynecki et al., 2020). Furthermore, no toxin or antibiotic resistance genes were identified, suggesting that these phages may be safe candidates for use as antibacterial agents in food applications (Barache et al., 2024).

#### 3.8.2 Comparative genomic analysis

**Figure 8:**
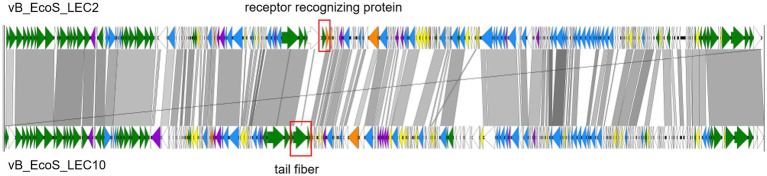
Pairwise genome comparison of phages LEC2 and LEC10. Whole-genome alignment illustrating pairwise comparisons between annotated open reading frames (ORFs) of phages LEC2 and LEC10. Regions of nucleotide sequence homology are indicated in gray, with darker shading representing higher sequence identity. Non-homologous regions are shown as gaps. The regions enclosed by rectangles indicate genes associated with receptor recognition.

To determine the specific characteristics of phages LEC2 and LEC10, their genomes were compared using whole-genome sequence alignment. A comparative analysis of the LEC2 and LEC10 genomes revealed that both share 74.6% sequence identity at the DNA level (Figure 8). It is worth noting that genomic comparison showed differences in genes related to receptor recognition and host specificity. Based on the previous results, differences were expected in the sequences of proteins involved in receptor recognition, as these phages recognize distinct host receptors. Tail spike proteins, tail fibers, and receptor-recognizing proteins play crucial roles in host recognition and determine phage specificity (Lee et al., 2016; Sørensen et al., 2021). ORF analysis of the LEC2 genome identified a receptor-recognizing protein that likely mediates interactions between LEC2 and its host. Comparative analysis revealed no sequence homology between the receptor-recognizing protein in LEC2 and tail fiber protein in LEC10, which is also involved in host recognition (Figure 8). The absence of sequence homology in genes responsible for receptor recognition further confirms that the two phages target different receptors, namely, OmpC and LPS. This finding provides molecular evidence that explains their distinct host ranges.

## 4 Conclusion

In this study, two novel lytic phages, LEC2 and LEC10, were isolated and characterized for their biocontrol potential against *E. coli*. The analysis confirmed their strong antibacterial activity, broad host range, and stability under various environmental conditions, supporting their potential application in the food industry. A phage cocktail composed of LEC2 and LEC10 demonstrated enhanced antibacterial efficacy compared to individual phages. The cocktail effectively lysed naturally contaminated, unidentified *E. coli* on fresh vegetables, suggesting its potential applicability in real-world food safety interventions. Receptor analysis revealed that LEC2 recognizes LPS (via WaaC), while LEC10 targets OmpC, confirming their distinct host recognition mechanisms and supporting their compatibility in a cocktail optimization. Genomic characterization further demonstrated the absence of lysogeny-related genes, toxins, and antibiotic resistance genes, reinforcing their safety for use in food applications.

Overall, these findings suggest that the LEC2-LEC10 phage cocktail could serve as a promising biocontrol strategy to reduce *E. coli* contamination in fresh produce. Further studies should investigate the scalability of phage applications in commercial food processing environments and the potential for combining additional phages to further broaden host coverage and minimize resistance development.

## Data Availability

The datasets presented in this study can be found in online repositories. The names of the repository/repositories and accession number(s) can be found in the article/Supplementary material.

## Footnotes

^1^ https://blast.ncbi.nlm.nih.gov/Blast.cgi/

^2^ https://www.gardp.org

^3^ https://www.uniprot.org/proteomes

^4^ http://mjsull.github.io/Easyfig/files.html

^5^ https://www.phage.ai/
